# A Reproducible Technique for Creation of the Subscapularis Split During Dynamic Anterior Stabilization for Shoulder Instability

**DOI:** 10.1016/j.eats.2020.06.007

**Published:** 2020-09-02

**Authors:** Mohamed Ibrahim, Pablo Narbona, Patrick J. Denard, Paolo Arrigoni, Philippe Collin, Alexandre Lädermann

**Affiliations:** a Department of Orthopaedics and Trauma Surgery, Faculty of Medicine, Fayoum University, Fayoum, Egypt; b Department of Shoulder Surgery, Sanatorio Allende, Córdoba, Argentina; c Department of Orthopaedic & Rehabilitation, Oregon Health & Science University, Portland, OR, USA; d La Clinica Ortopedica, ASST PINI-CTO, Università degli Studi di Milano, Milan, Italy; e Centre Hospitalier Privé Saint-Grégoire (Vivalto Santé), Saint- Grégoire, France; f Division of Orthopaedics and Trauma Surgery, La Tour Hospital, Meyrin; g Faculty of Medicine, University of Geneva, Switzerland; h Division of Orthopaedics and Trauma Surgery, Department of Surgery, Geneva University Hospitals, Geneva, Switzerland

## Abstract

The subscapularis split is a required difficult step of several instability procedures. We propose creating a subscapularis split using the shuttled long head of the biceps by simple passive external rotation of the arm during dynamic anterior stabilization. This technique simplifies one of the technically demanding steps of dynamic anterior stabilization, making the split quicker and more reproducible.

Dynamic anterior stabilization (DAS) of the shoulder seems capable of closing the gap between the indications for isolated Bankart repair and bone-transfer techniques, as it combines a Bankart repair with the additional sling effect of the long head of the biceps (LHB) tendon of Latarjet procedures to treat anterior glenohumeral instability.1 Biomechanically, the DAS technique leads to a significantly decreased anterior glenohumeral translation in the context of small anterior glenoid bone defects at the time of surgery.2 Clinically, this may reduce the risk of a conflict between the humeral head and the anterior margin of the glenoid, leading to more secure stable shoulder than with isolated Bankart repair with a less-invasive approach with fewer complications than Latarjet procedures.

The subscapularis split is a required difficult step of several instability procedures such as DAS,^1^^,^^2^ Latarjet,^3^, ^4^, ^5^ Eden–Hybinette,^6^^,^^7^ or open bony Bankart fracture fixation. The arthroscopic creation of such split is particularly challenging and has been seen as a challenge in the learning curve of arthroscopic bone block procedures or DAS.

The subscapularis split can be performed with several variations: before^4^ or after the coracoid osteotomy,^3^^,^^5^ with inside-out or outside-in approaches, using a knife then scissors to spread the muscle (a common open technique), or an electrocautery to burn the muscle fibers (a common arthroscopic technique). The level of the split can be difficult to establish.8 The proximity of the axillary nerve and brachial plexus makes its creation inherently risky. Furthermore, it is the natural tendency to begin the split laterally, in the robust tendinous part of the subscapularis, even if the aim should be to spread only the medial and fragile muscular fibers. Consequently, development of a safe and reproducible technique to perform the split, particularly arthroscopically, is appealing.

This article describes a technique of subscapularis split using the shuttled LHB by simple passive external rotation of the arm during DAS, with desired effective length and without additional dissection.

## Surgical Technique

### Preoperative Patient Positioning

The operation is performed with the patient in the semi-beach chair position under general anesthesia with an interscalene block. Table 1 explains the risks and/or limitations of this technique.

**Table 1:** Pearls and Pitfalls

Pearls
The subcoracoid spaced should be thoroughly cleared to prepare for LHB transfer.
Open the bicipital groove first laterally and distally to avoid detaching the subscapularis.
After passing it into the glenohumeral joint, maintain tension on the LHB tendon by pulling sutures from posterior portal when performing rotation of the arm to spread the muscle fibers of the subscapularis.
Pitfalls
Difficult passage of the graft through the subscapular muscle in case of insufficient horizontal split of the capsule.
Damage to the nerve plexus if the retrograde suture passage introduced through the posterior portal is not maintained lateral to the conjoint tendon during subscapular split or if not pushed through the subscapularis muscle under visual control.

LHB, long head of the biceps.

### Initial Exposure and Portal Placement

**Fig 1:**
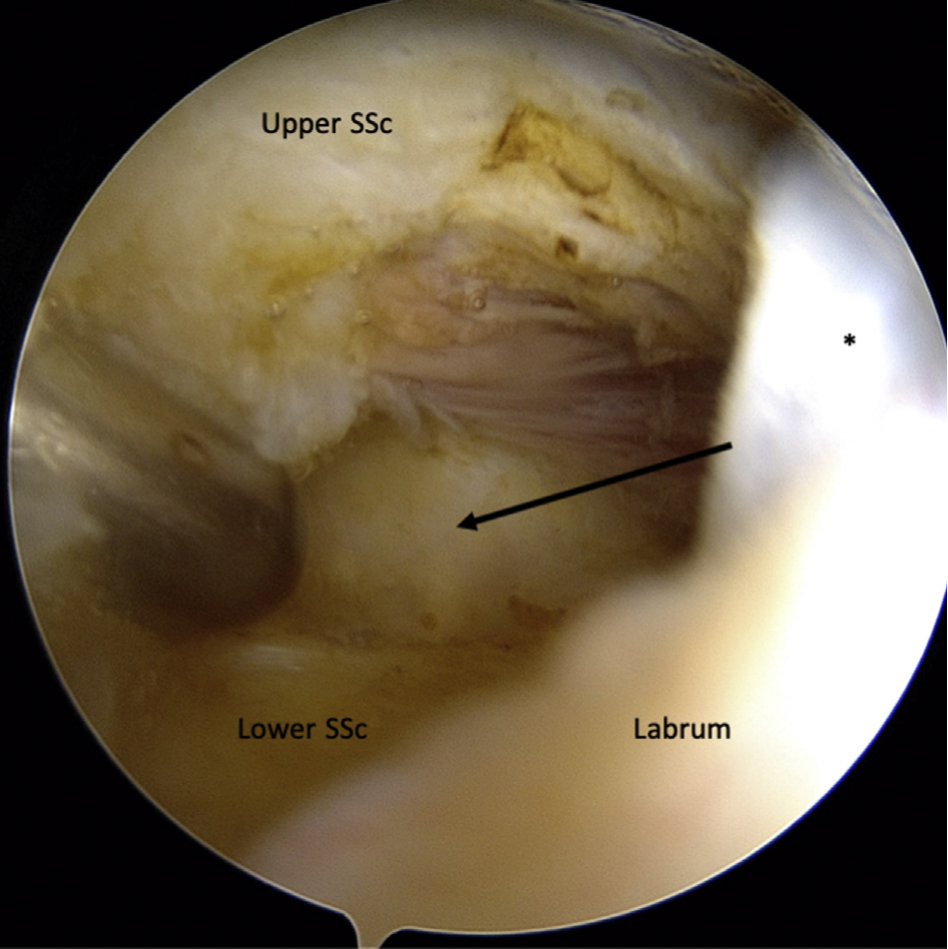
Intra-articular view of a left shoulder, standard posterior viewing portal. ASL portal has been established by an outside-in technique using a spinal needle as a guide. The rotator interval has been widely opened. A horizontal split (arrow) in the capsule is performed with an electrocautery coming from the ASL portal. ∗Middle glenohumeral ligament. (ASL, anterosuperolateral; SSc, subscapularis.)

An intra-articular approach is used through a standard 3-portal (posterior, anterosuperolateral [ASL], and anterior portals) technique. The posterior portal is established at the “soft spot” 2 cm inferior and 1 cm medial to the posterolateral edge of the acromion. A standard diagnostic arthroscopy is performed with a 30° arthroscope and a pump maintaining pressure at 40 mm Hg. ASL and anterior working portals are then established with an outside-in technique using a spinal needle as a guide. The rotator interval is opened with an electrocautery to visualize the superior tendinous border of the subscapularis for later measurement and instrument access. From the ASL portal, the capsule posterior to the subscapularis muscle is horizontally opened with electrocautery (Fig 1). A 70° measurement probe is used to mark the intended split at approximately 35 mm in male patients and 30 mm in female patients inferior to the upper border of the subscapularis tendon.^8^

The arthroscope is then advanced anteriorly through the window in the rotator interval and the subcoracoid space is cleared to obtain complete visualization of the anterior deltoid, the subscapularis on 3 sides, the lateral margin of the conjoint tendon, and the pectoralis major. A mark is made on the LHB at the level of the pectoralis major for subsequent measurement.

### Addressing the LHB

**Fig 2:**
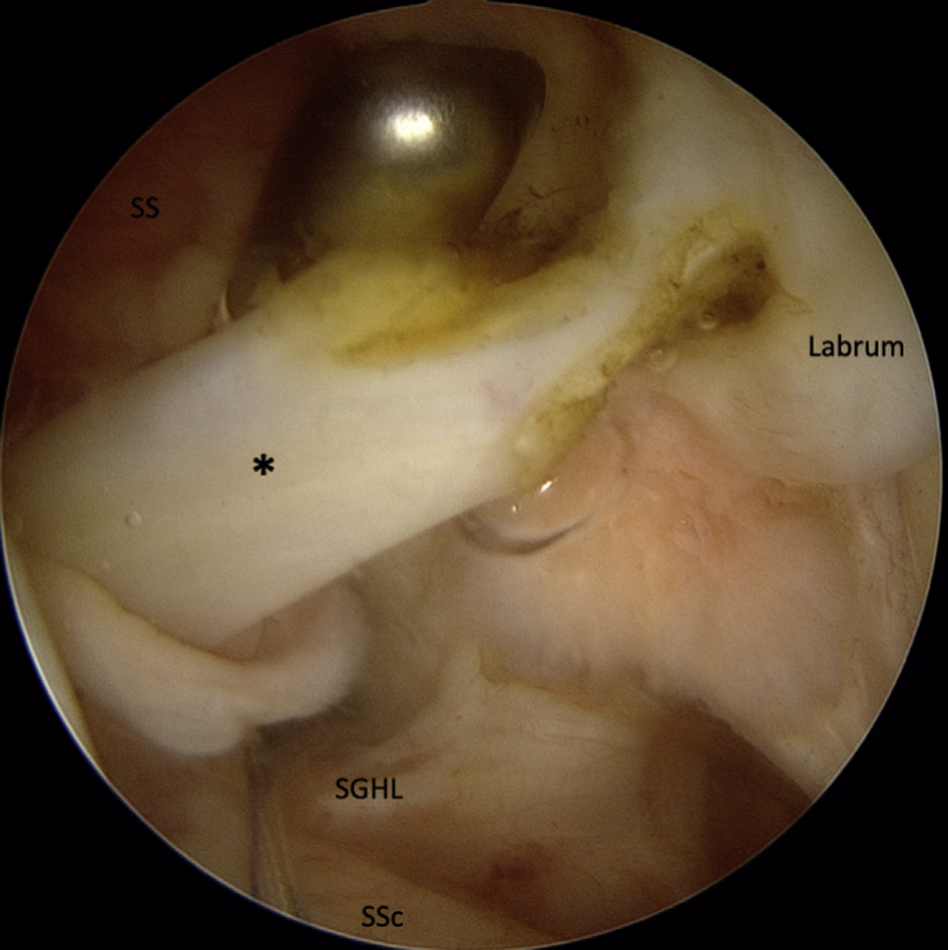
LHB (∗) tenotomy at its origin on the upper glenoid rim. (LHB, long head of the biceps; SGHL, superior glenohumeral ligament; SS, supraspinatus; SSc, subscapularis.)

**Fig 3:**
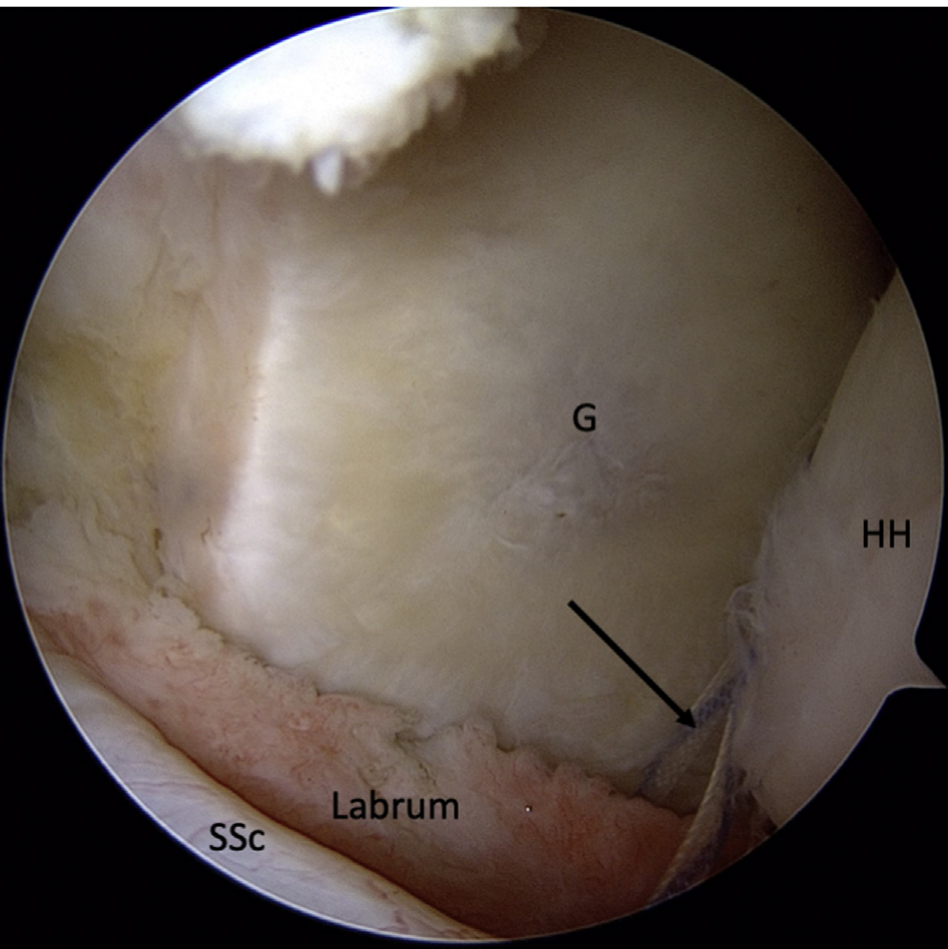
Intra-articular view of a left shoulder, ASL viewing portal. A suture (arrow) is passed around the detached labrum and pulled through the posterior portal to increase access for preparation of the anterior glenoid (G). (ASL, anterosuperolateral; HH, humeral head; SSc, subscapularis.)

**Fig 4:**
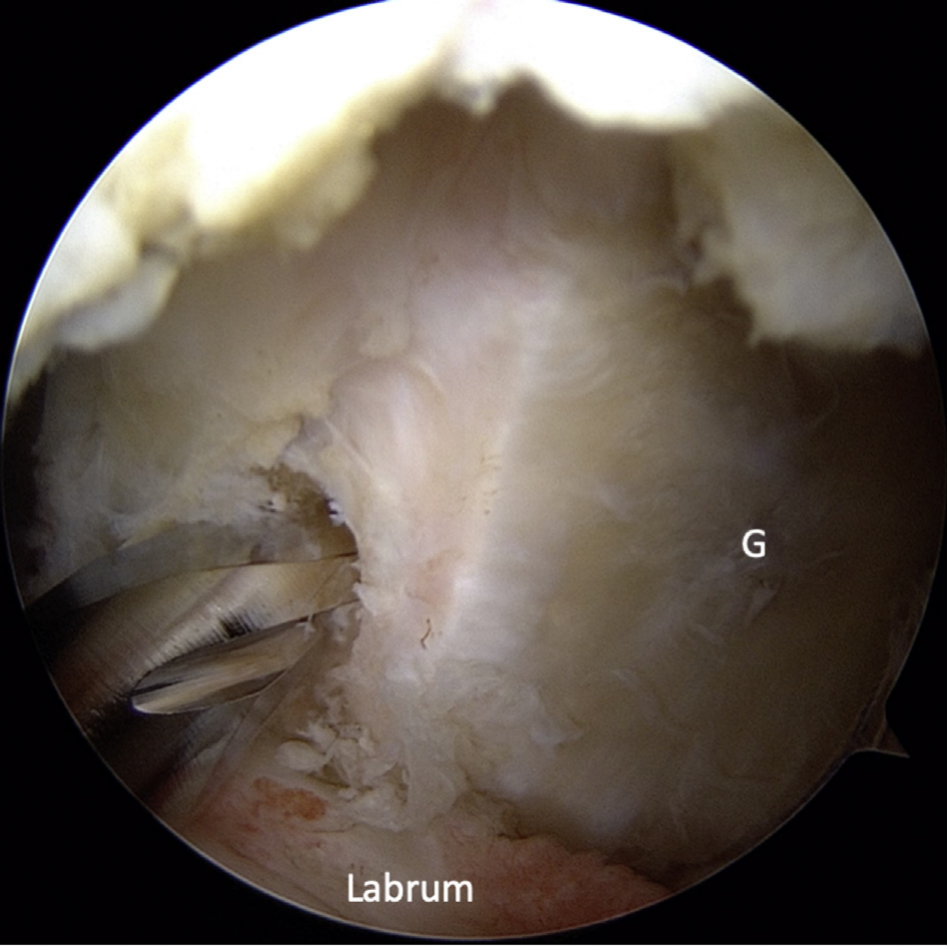
Intraoperative view of a left shoulder through the rotator interval, ASL viewing portal. A drill is used to prepare a bone socket at approximately 8 o'clock. The bone socket is oriented from anterior to posterior, lateral to medial, and superior to inferior. A 4-mm socket is created for a depth of 20 mm. (ASL, anterosuperolateral; G, glenoid.)

**Fig 5:**
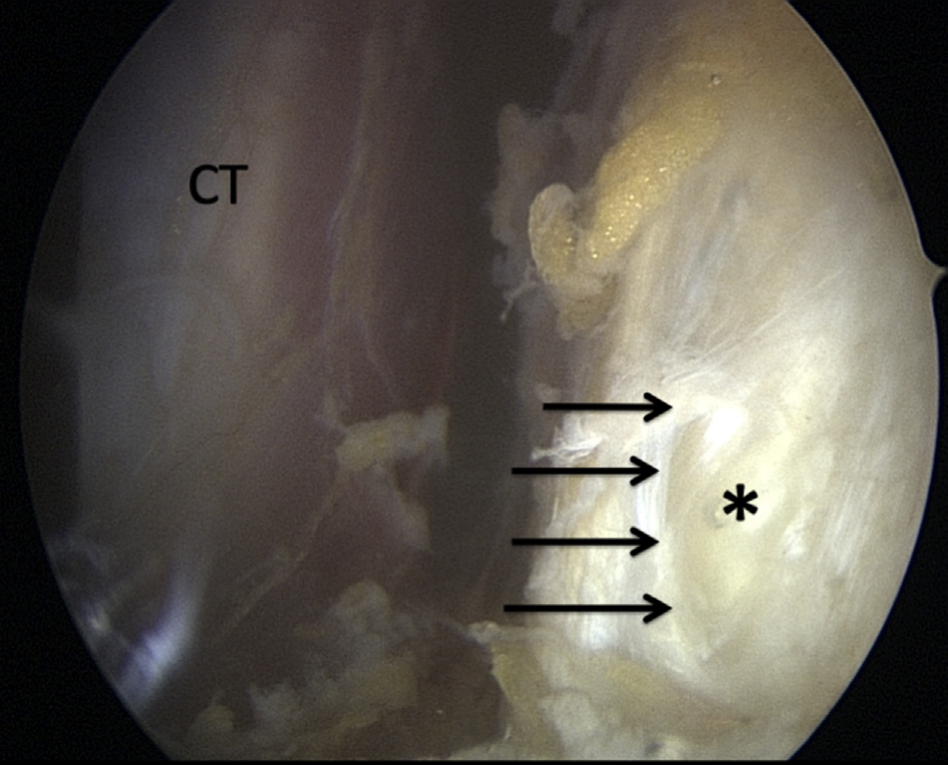
Subcoracoid space of a left shoulder, ASL viewing portal. After LHB tenotomy, the bicipital sheath (black arrows) is opened laterally and the LHB (∗) is found. (ASL, anterosuperolateral; CT, conjoint tendon; LHB, long head of the biceps.)

**Fig 6:**
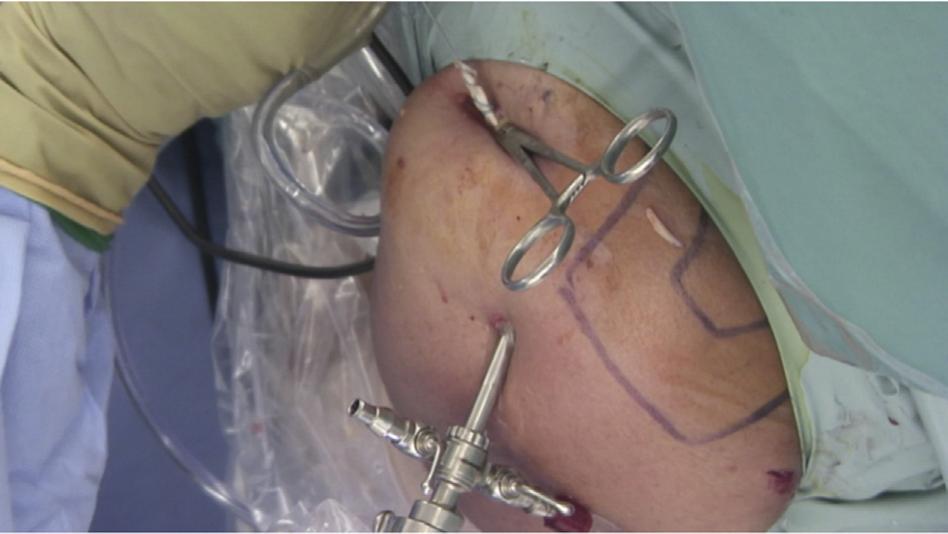
Superior view of a left shoulder. The LHB is exteriorized. Its diameter was decreased to 4 mm diameter for the proximal 2 cm. The 3 proximal centimeters of the proximal tendon were then secured with a whipstitch. (LHB, long head of the biceps.)

**Fig 7:**
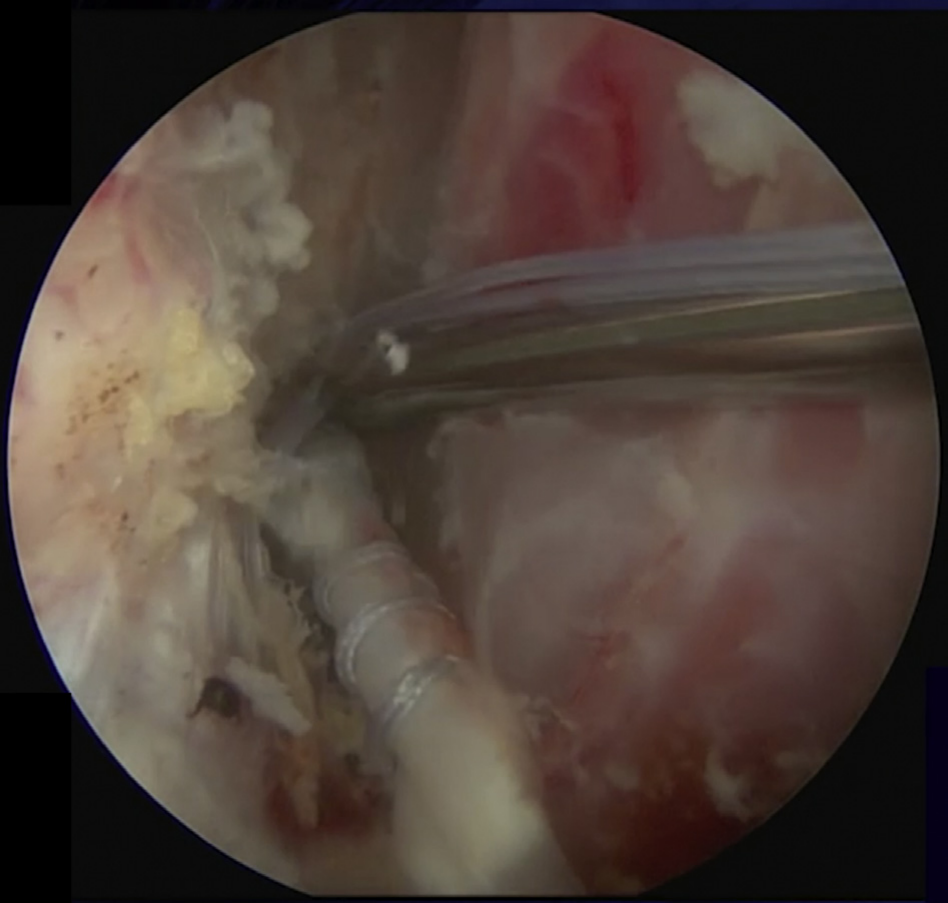
Subcoracoid view of a left shoulder from an ASL viewing portal. The LHB is pushed between the SSc and the CT. (ASL, anterosuperolateral; CT, conjoint tendon; LHB, long head of the biceps; SSc, subscapularis.)

The LHB is then tenotomized at its origin with curved arthroscopic scissors (Fig 2). The arthroscope is then moved to the ASL portal. A suture is passed around the detached labrum and pulled through the posterior portal to increase access for preparation of the anterior glenoid (Fig 3). A drill is used to prepare a hole at approximately 4 o'clock from

anterior to posterior, from lateral to medial and from superior to inferior, within the neck of the glenoid (Fig 4). The bicipital groove is opened laterally and distally to avoid detaching the subscapularis (Fig 5).^9^ A suture grasper is introduced through the anterior portal and the LHB is exteriorized and prepared (Fig 6). The 2 first cm are reduced to 4 mm of diameter. The proximal 3 cm of the tendon is whipstitched using a SpeedWhip (Arthrex, Naples, FL) technique with a No. 2 FiberLoop (Arthrex). The LHB is then pushed medially between the subscapularis and the conjoint tendon (Fig 7).

### Subscapularis Split Technique (With Video Illustration)

**Fig 8:**
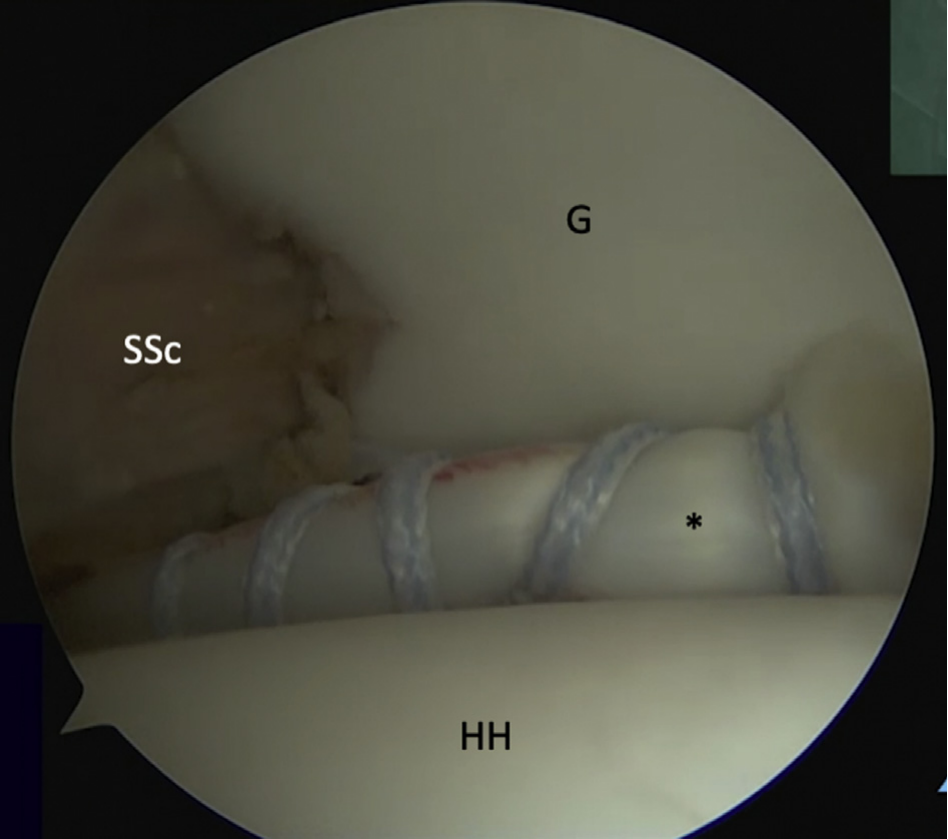
Intra-articular view of a left shoulder, ASL viewing portal. The LHB (∗) tendon has been shuttled through the SSc into the glenohumeral joint. (ASL, anterosuperolateral; G, glenoid; HH, humeral head; LHB, long head of the biceps; SSc, subscapularis.)

While viewing from the ASL portal, a retrograde suture passage is introduced through the posterior portal and through the subscapularis at the level of the previously marked split location (Video 1, reproduced from https://wiki.beemed.com, with permission). The retrograde passer is used to retrieve the ends of the LHB whipstitch sutures and then pull the LHB tendon into the glenohumeral joint (Fig 8).

**Fig 9:**
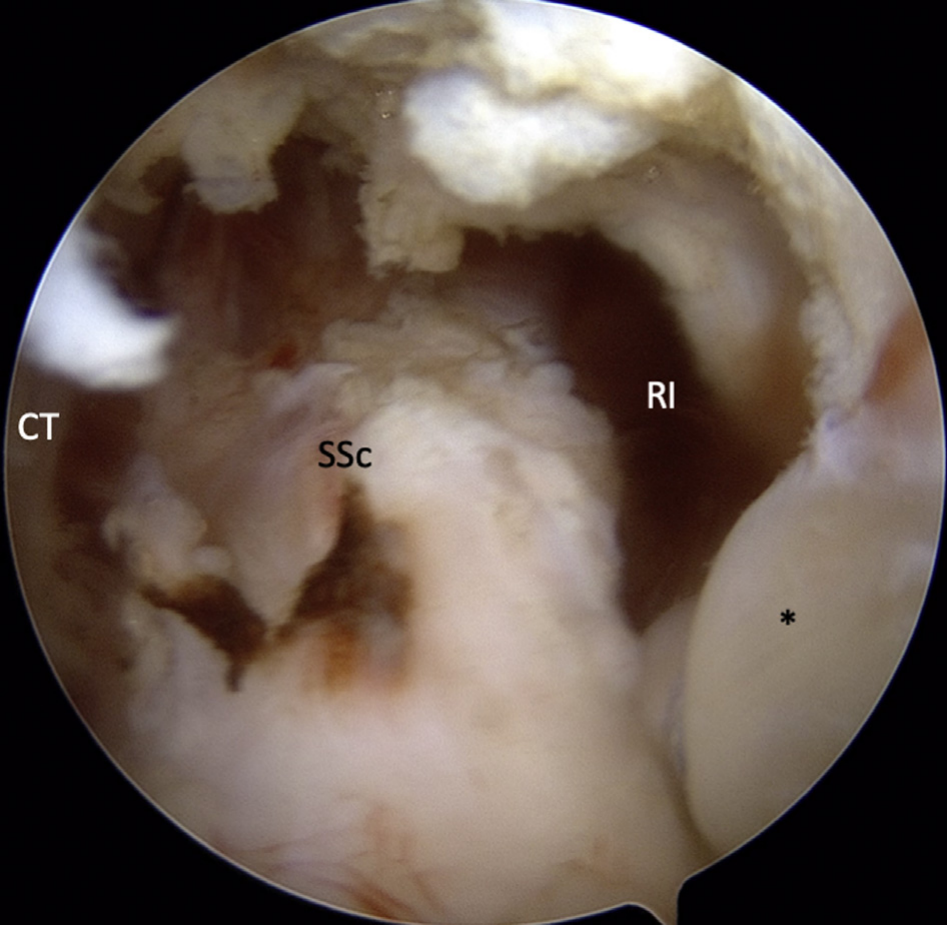
Intra-articular view of a left shoulder, ASL viewing portal. The LHB (∗) passes through the SSc split into the glenohumeral joint and is brought back to anterior portal through the RI to load the interference screw. (ASL, anterosuperolateral; CT, conjoint tendon; LHB, long head of the biceps; RI, rotator interval; SSc, subscapularis.)

**Fig 10:**
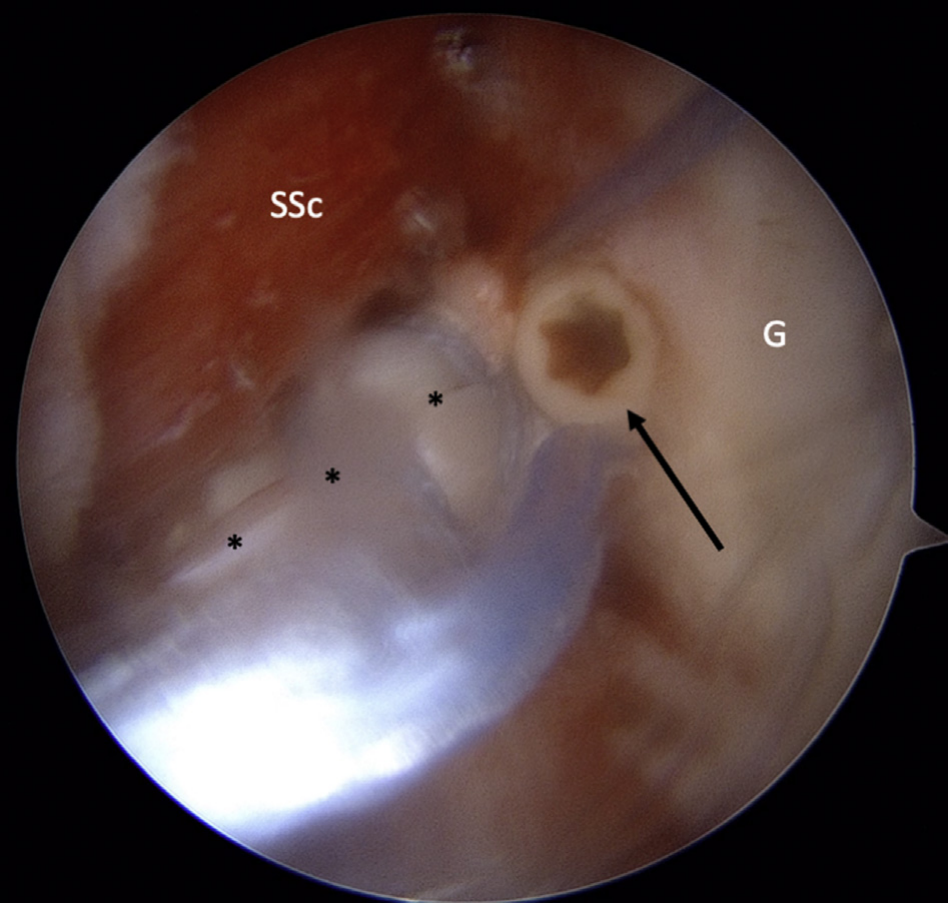
Intraoperative view of a left shoulder through the rotator interval, ASL viewing portal. The LHB tendon (∗) is pushed back through the rotator interval into the predrilled hole and secured onto the glenoid using a tenodesis screw (arrow). (ASL, anterosuperolateral; G, glenoid; LHB, long head of the biceps; SSc, subscapularis.)

**Fig 11:**
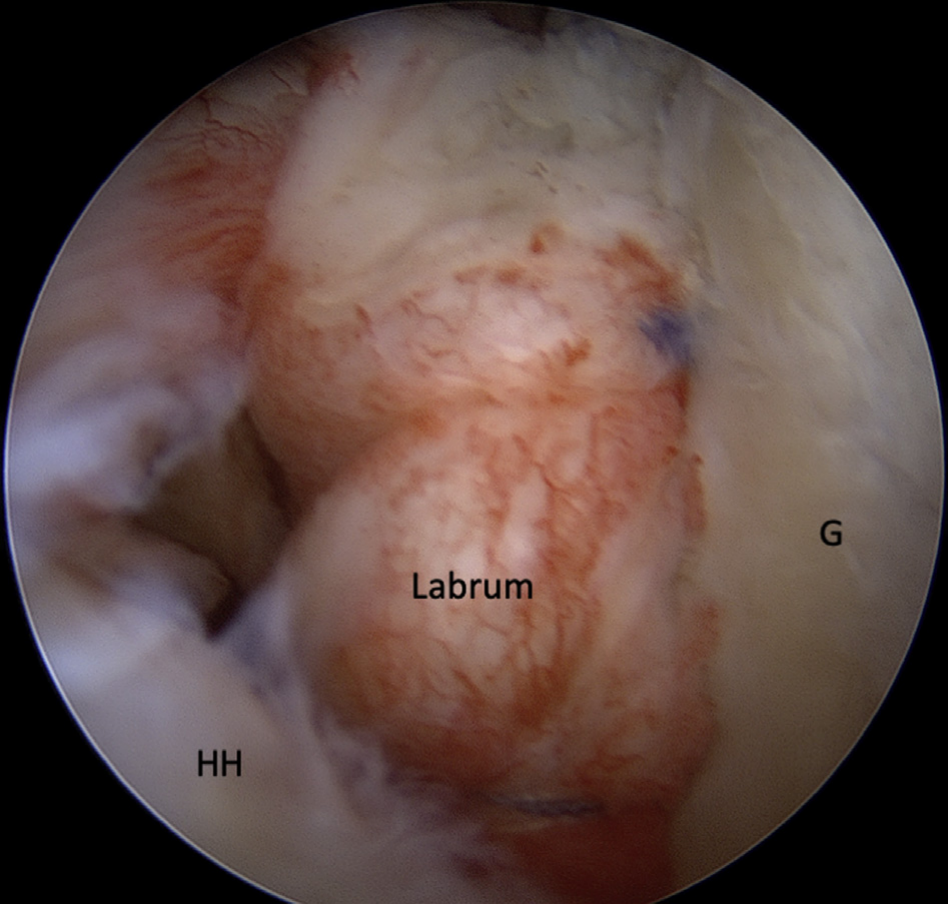
Intra-articular view through the posterior portal. A standard Bankart repair is performed using 3 suture anchors. The anchors are placed on the glenoid rim at 9, 8, and 7 o'clock (3, 4, and 5 o'clock for a right shoulder) to enable tensioning of the capsuloligamentous structures and to re-establish the labral bumper effect. (G, glenoid; HH, humeral head.)

With the LHB passed through the desired split level, the arm is then passively maximally externally and internally rotated to spread the muscle fibers of the subscapularis (Video 2, reproduced from https://wiki.beemed.com, with permission). The LHB is brought back to anterior portal through the rotator interval to load the interference screw (Fig 9). The LHB is then fixed on the glenoid at 4 o'clock with a knotless anchor (3.9 SwiveLock; Arthrex) (Fig 10). A standard Bankart repair is finally performed using 3 suture anchors (Fig 11).

### Postoperative Rehabilitation

Patients are instructed to wear a simple sling for 10 days to encourage rest and reduce the risk of postoperative hematoma. Rehabilitation with self-mobilization in elevation and external rotation is allowed from day 0. At 10 days postoperatively, activities of daily living are allowed and self-mobilization in elevation and external rotation are continued. Return to low-risk sports (e.g. jogging, cycling, and swimming) is allowed at 6 weeks, and high-risk (throwing and collision) sports at 3 months only after satisfactory clinical evaluation.

## Discussion

It is not necessary to create a distinct large split in the subscapularis tendon before shuttling the LHB into the glenohumeral joint for DAS. This finding has several implications relevant to the DAS and arthroscopic creation of a subscapularis split.

Similarity between arthroscopic Latarjet and DAS procedure relies upon shuttling of coracoid bone block or LHB, respectively, through subscapularis split. Previous reports on arthroscopic Latarjet stabilization procedures have described the creation of a distinct wide subscapularis split via electrocautery before shuttling into the glenohumeral joint.^5^ This step increases the technical difficulty of the procedure and requires additional work around the axillary nerve and brachial plexus. This step may partially explain the 1% rate of neurologic injury following arthroscopic Latarjet.^10^ In contrast to the Latarjet in which a large bone block is shuttled into the joint, with the DAS only the LHB is shuttled into the joint, as this procedure relies upon the sling effect only. Clinically we observed that the LHB was easy to shuttle into the joint without creation of a distinct split and that subsequent passive external rotation created the split. The present study confirms these observations. A significant split length can be obtained with passive external and internal rotation of the arm. This technique is appealing in that it is less invasive than the active creation of a split. Furthermore, the necessary length of the split can be patient dependent in that is will vary based on each individual's maximal external rotation. This technique may also be adapted to arthroscopic Latarjet.

DAS relies on the LHB tendon, which has a smaller diameter than the conjoint tendon, and could therefore have weaker “hammock” and “sling” effects^11^ than that of the standard Latarjet. As with the Latarjet procedure, there is risk of postoperative biceps pain. Furthermore, the DAS does not involve bony reconstruction and may therefore be inadequate for cases with substantial glenoid bone loss.^2^ Table 2 summarizes the advantages and disadvantages of this technique.

**Table 2:** Advantages and Disadvantages

Advantages
Easier, safer, and less invasive than conventional active creation of a split.
Maintains the important “hammock” and “sling” effects to Bankart repair.
Avoid nerves dissection, axillary nerve protection, or retraction.
The entire procedure can be performed through the rotator interval.
Disadvantages
Depending of suppleness, limited spread the muscle fibers of the subscapularis after maximal rotation of the arm elbow at side.
No long-term follow-up.

LHB, long head of the biceps.

## Conclusions

A subscapularis split can be created by passive external rotation of the arm after the LHB is shuttled into the joint during DAS. Therefore, there is no need to create a distinct split before DAS.

## Supplementary Data

Video 1 Arthroscopic subscapularis split technique. Intra-articular view of a left shoulder from a posterior viewing portal. A probe is inserted through the ASL portal. A horizontal split in the capsule is created until the muscle fibers of the subscapularis are seen. While viewing from the ASL portal, a retrograde suture passer is introduced through the posterior portal and through the subscapularis at the level of the previously marked capsular split location. The LHB and the whipstitch sutures are then pushed between the conjoint tendon and the subscapularis. Under direct visualization, the retrograde passer is pushed through the subscapularis muscle, to retrieve the whipstitch sutures and pull the LHB tendon into the glenohumeral joint. The arm is then passively maximally externally and internally rotated to spread the muscle fibers of the subscapularis. (ASL, anterosuperolateral; LHB, long head of the biceps.)

ICMJE author disclosure forms

Video 2 Open demonstration of the split. Passive external and internal rotation of the arm is performed to automatically spread the muscle fibers of the subscapularis.
